# Pericardial Fat Relates to Disturbances of Glucose Metabolism in Women with the Polycystic Ovary Syndrome, but Not in Healthy Control Subjects

**DOI:** 10.1155/2018/5406128

**Published:** 2018-08-07

**Authors:** Michael Leutner, Christian Göbl, Peter Wolf, Katharina Maruszczak, Latife Bozkurt, Helmut Steinbrecher, Ivica Just-Kukurova, Johannes Ott, Christian Egarter, Siegfried Trattnig, Alexandra Kautzky-Willer

**Affiliations:** ^1^Department of Internal Medicine III, Clinical Division of Endocrinology and Metabolism, Unit of Gender Medicine, Medical University of Vienna, Waehringer Guertel 18-20, 1090 Vienna, Austria; ^2^Department of Obstetrics and Gynecology, Division of Gynecologic Endocrinology and Reproductive Medicine, Medical University of Vienna, Vienna, Austria; ^3^Department of Internal Medicine III, Clinical Division of Endocrinology and Metabolism, Medical University of Vienna, Waehringer Guertel 18-20, 1090 Vienna, Austria; ^4^Department of Biomedical Imaging and Image-guided Therapy, Centre of Excellence-High Field MR, Medical University of Vienna, Vienna, Austria

## Abstract

**Objective:**

The objective of the present study is to investigate the relationship of cardiac fat depots with disturbances of the carbohydrate metabolism in women with PCOS.

**Methods:**

An oral glucose tolerance test (OGTT) was realized, and metabolic parameters were collected in 48 women with PCOS and in 20 controls. Intramyocardial fat (MYCL) and pericardial fat (PERI) were measured using ^1^H-magnetic resonance spectroscopy and imaging.

**Results:**

Only in PCOS women, PERI was positively and independently related to parameters of glucose metabolism (HbA1c: *p* = 0.001, fasting plasma glucose: *p* < 0.001, stimulated glucose at 30 and 60 minutes in the OGTT). Thus, the disposition index, insulin sensitivity, and adiponectin also declined with the increase of PERI in women with PCOS; however, these results were not independent of BMI and age. In addition, PERI was positively related to atherogenic lipid profiles, BMI, waist circumference, CRP, and liver fat in women with PCOS. A negative relation of PERI with triglycerides and a positive relation with BMI and waist circumference could be observed in the controls. No relationship of MYCL with diabetes-specific parameters could be found in the study population.

**Conclusion:**

PERI is related to metabolic disturbances in women with PCOS, but not in metabolically healthy lean subjects. This clinical trial was registered at ClinicalTrials.gov and has the registration number NCT03204461.

## 1. Introduction

The polycystic ovary syndrome (PCOS) is a common endocrine disorder [1] and has been shown to affect 5–10% of women at childbearing age [2]. Common disturbances of carbohydrate metabolism in PCOS are insulin resistance and hyperinsulinemia [3]. Compared to healthy women in the reproductive age, women with PCOS are at high risk for developing type 2 diabetes [1]. It has been shown that women with PCOS in the United States have a tenfold higher risk for developing type 2 diabetes compared to healthy women at the same age. In addition, they are characterised as having a two to three times higher prevalence rate of the metabolic syndrome when compared to healthy control subjects [2]. Insulin resistance plays a major role in the occurrence of an anovulatory state in women with PCOS [2]. Therefore, it has been shown that metformin has significant positive effects on this relationship [4]. However, the underlying pathophysiological mechanisms of the increased risk for type 2 diabetes and therefore especially for insulin resistance are not yet fully understood in women with PCOS [2]. Several study groups investigated these mechanisms and could show that there is a close relationship of the amount of liver fat with insulin resistance in women with PCOS [5, 6]; however, a recent study could not find a difference in the amount of liver fat between women with PCOS and control subjects [7]. Several study groups investigated the impact of cardiac fat depots and demonstrated that there is a relationship to disturbances of glucose metabolism [8–10]. However, especially data about the role of cardiac fat depots in glucose metabolism in women with PCOS is still controversial [11–15], although it has been shown that there is a relationship of epicardial fat (EPI) with visceral abdominal adipose tissue [11]. Further, earlier studies could show that EPI is an endocrine active organ that expresses antidiabetogenic factors such as adiponectin [16] and that the fat content surrounding the heart is related to metabolic disturbances [10, 17, 18]. However, the fact that the pathophysiological mechanisms of an increased metabolic risk in women with PCOS is not clear so far and that the fat content around the heart has not yet been investigated thoroughly in this specific population led to the following aim: to investigate the relationship of PERI and MYCL with disturbances of glucose metabolism (i.e., insulin resistance; beta cell compensation in insulin resistance; whole body insulin sensitivity; dynamic changes of glucose, insulin, and C-peptide levels during the oral glucose tolerance test “OGTT” and insulin secretion) in women with PCOS.

## 2. Materials and Methods

The detailed description of the study procedures of this prospective cross-sectional study was explained previously [7]. However, in the present analysis, only participants who had a magnetic resonance spectroscopy (MRS) of the heart were included. Thus, in the analysis of [7], also participants who did not have a MRS of the heart were included. In short, 48 women who were diagnosed with PCOS and who were untreated, as well as 20 control subjects, had a cardiac magnetic resonance (MR) spectroscopy for the measurement of MYCL. PERI was analyzed in 43 women with PCOS (NIH group: *n* = 26, ROT group: *n* = 17) and in 20 control subjects based on a four-chamber orientation image. The study group of women with PCOS was built up according to the criteria of the National Institute of Health (NIH), which included 31 patients, and according to the Rotterdam criteria, which included 17 patients. Diabetes mellitus type 2, antidiabetic drugs, dyslipidemic treatment, and other diseases, which influence the reproductive system, were excluded. The control group consisted of 20 healthy women (10 women in the control group had a systemic hormonal contraceptive treatment) [7]. The local ethics committee of the Medical University of Vienna approved the study protocol, which was performed in accordance with the Declaration of Helsinki.

### 2.1. Laboratory Measurements

For the exact assessment of the glucose metabolism, a 75 g OGTT, with the measurement of fasting plasma glucose, fasting insulin, and fasting C-peptide at 0, 30, 60, 90, and 120 minutes, was done. In addition, lipid parameters, hormones, and anthropometric data, such as BMI and waist circumference, were assessed. Laboratory measurements were assessed after a >8-hour fasting period. The analyzation of the laboratory parameters was accomplished at the certified Department of Medical and Chemical Laboratory Diagnostics of the Medical University of Vienna (http://www.kimcl.at) [7]. Adiponectin was analyzed with a competitive enzyme immunoassay of BioVendor (BioVendor Human Adiponectin ELISA).

### 2.2. Measurements of Ectopic Lipids

Myocardial lipid content (MYCL) was measured according to previously described standardized procedures, on a 3.0 Tesla Magnetom Trio Siemens System [19, 20].

Pericardial fat (PERI), which is the sum of epicardial and paracardial adipose tissue and which is located around the heart, was also analyzed according to previous studies [10, 19, 21, 22]. Therefore, a four-chamber orientation image was used for the analysis of pericardial adipose tissue by T1-weighted ECG-gated cine true fast imaging. Three slices (from the apex to the pulmonary trunk) were used for the drawing around the edges of the adipose tissue of the heart. At the end, the average value of pericardial fat, which was measured in the three slices, was given in cm^2^ [19].

The amount of ectopic lipids in the liver was also measured on a 3.0 Tesla Magnetom Trio Siemens System, according to standardized and previously described methods [7].

### 2.3. Calculations

The Matsuda index for the evaluation of total body insulin sensitivity [23] and the oral glucose insulin sensitivity index (OGIS) [24] were calculated with data of the OGTT. For the assessment of hepatic insulin resistance, HOMA-IR was used [25]. The insulinogenic index was used for the assessment of insulin secretion during the OGTT [26]. The oral disposition index (assessment of beta cell function in insulin resistance) was calculated as the product of the insulinogenic index and the Matsuda index. The diagnosis of the metabolic syndrome was defined according to the criteria of the National Cholesterol Education Program (NCEP-ATP-III).

### 2.4. Statistical Analysis

Medians and interquartile ranges were applied for the presentation of continuous variables. Mann–Whitney-*U* test was used for group-based comparisons and Spearman correlation rank test for correlation analyses because normality assumptions of the variables were violated. The nonparametric Kruskal-Wallis test was used for the assessment of differences between more than two groups. Linear regression models were utilized for multivariable adjustment including BMI and age. Therefore, all results which were not independent of BMI and age were stated in the manuscript. A two-sided *p* value of <0.05 was considered as statistically significant. Statistical analysis was performed with IBM SPSS version 23.

## 3. Results

### 3.1. Baseline Clinical and Metabolic Characteristics

The detailed baseline characteristics of the study population in the present study were previously published [7]. In this analysis, 31 women with PCOS, defined according to the NIH criteria, and 17 women with PCOS defined according to the ROT criteria, as well as 20 control subjects, were included.

Table 1 shows the baseline characteristics including anthropometric and clinical data, as well as metabolic and endocrine parameters. Women with PCOS showed a worse metabolic profile such as higher BMI levels, increased waist circumference measures, lower HDL cholesterol, and higher triglyceride levels and were characterised by a more pronounced insulin resistance compared to the control group (Table 1). In detail, one study participant in the PCOS-NIH group had an impaired glucose tolerance (IGT), while no participant suffered from impaired fasting glucose (IFG) in the present study. Seven participants in the PCOS-NIH group and four participants in the PCOS-ROT group were shown to be insulin resistant (defined by a HOMA-IR ≥ 2.5). The metabolic syndrome occurred in two women, one being in the PCOS-NIH group and the other in the PCOS-ROT group.

### 3.2. PERI

Considering the amount of PERI, no significant difference can be reported between the group of women with PCOS and the group consisting of healthy women (Table 1).

#### 3.2.1. PERI and Glucose Metabolism

**Figure 1:**
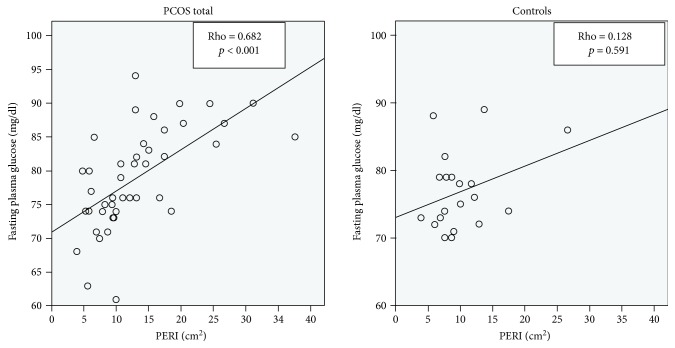
Correlation analyses of PERI with fasting plasma glucose in PCOS women and control subjects.

Correlation analyses showed that only in women with PCOS, a positive relation of PERI with parameters of glucose metabolism, including the following, could be observed: HbA1c (Table 2) and fasting plasma glucose (Figure 1 and Table 2), as well as dynamic glucose levels after 30, 60, 90, and 120 minutes in the OGTT (Table 2). A negative relation of PERI with the beta cell function in insulin resistance (measured with the disposition index) and with insulin sensitivity (measured with OGIS) was found in women with PCOS (Table 2). Additional statistical analyses showed that there is a negative relationship of PERI with the levels of adiponectin (rho = −0.39, *p* = 0.009) in the PCOS total group, which was not independent of BMI. Thus, adiponectin was negatively related to insulin resistance which was evaluated with the HOMA-IR (rho = −0.33, *p* = 0.032) in the PCOS total group. However, this relationship was not independent of the BMI. Linear regression models showed that all the observed significant relationships of PERI with parameters affecting glucose metabolism still remained significant after the correction for BMI and age, except glucose levels at 90 and 120 minutes in the OGTT, the disposition index, OGIS (Supplementary Table 1), and dynamic insulin levels at 60 minutes. By splitting the study cohort of women with PCOS, according to the NIH and the ROT criteria, all significant relationships of PERI with parameters of glucose metabolism were independent of BMI and age, except glucose levels at 30 and 120 minutes in the NIH group. As shown in Table 2 and in contrast to the PCOS group, there was no correlation of PERI with parameters and indices of glucose metabolism in the control group. These missing relationships were also observed in a sensitivity analysis, after excluding 10 women with systemic contraceptive agents.

### 3.3. PERI and Ectopic Lipids

As displayed by Table 3, there was an association of PERI with the lipid content in the liver in women with PCOS.

### 3.4. PERI and the Relation to Lipid Parameters and to Parameters of Body Composition

In women with PCOS, a positive relation of PERI with LDL cholesterol and a negative one with HDL cholesterol could be observed, yet these relations were not independent of BMI and age (Table 3). In control subjects, there was a negative association of PERI with triglyceride levels (Table 3). PERI was significantly related to parameters of body composition, such as BMI and waist circumference in control subjects, as well as in women with PCOS (Table 3).

### 3.5. PERI and Hormones

Control subjects showed a significant and positive correlation of PERI with prolactin levels (Table 3). Therefore, the correlation of PERI with prolactin levels in the control group remained significant in a sensitivity analysis, after the exclusion of 10 women with systemic contraceptive agents. However, neither in the control group nor in the PCOS group, a relationship of PERI with estradiol, testosterone, progesterone (Table 3), or the free androgen index (FAI) could be observed.

### 3.6. MYCL

As shown in Table 1, no difference in the amount of MYCL between women with PCOS and control subjects was observed in this study. Neither in women with PCOS nor in the control subjects, a relation of MYCL to parameters of glucose metabolism (HbA1c, insulin, fasting glucose and stimulated glucose, C-peptide, Matsuda index, HOMA-IR, insulinogenic index, and OGIS), lipid parameters (triglycerides, LDL cholesterol, HDL cholesterol, and total cholesterol), blood pressure, heart frequency, inflammatory parameter CRP and us-CRP, cortisol, hormones, and ectopic lipids could be reported.

## 4. Discussion

In the present study, we investigated the impact of PERI and MYCL on basal and dynamic levels of parameters of carbohydrate metabolism in women with PCOS. Women with PCOS, especially when defined by the NIH criteria, are characterised by having an inauspicious body composition, as well as having worse levels of diabetes specific parameters when compared to healthy control subjects. The present study shows that only in PCOS women, PERI is related to parameters of glucose metabolism. Women with PCOS also feature a decreased beta cell compensation in insulin resistance, as well as a decreased insulin sensitivity, alongside an increase of PERI. However, we could not find a relationship of the intramyocardial lipid content with diabetes-specific parameters. According to these results, one can hypothesize that PERI, but not MYCL, is involved in the pathophysiological mechanisms of disturbances in glucose metabolism in PCOS and may have positive effects under physiological conditions.

Women with PCOS are at an increased risk of developing metabolic diseases; however, the exact pathophysiological mechanisms of a pathological glucose metabolism are up to date not entirely known in this specific population [7]. There is some evidence that the amount of ectopic lipids, such as liver fat, may be significantly influential in the development of insulin resistance in women with PCOS [5, 6, 27]. Therefore, especially the role of EPI, which is an endocrine active organ and which is localized around the heart, is interesting in this specific population. Thus, data showed that EPI strongly expresses factors, which are related to disturbances of glucose metabolism (e.g., adiponectin), yet depending on physiological and pathological conditions [10, 16, 28]. The data about the role of the lipid content around the heart is sparse in women with PCOS. Studies showed that women with PCOS, diagnosed according to the Rotterdam criteria, are characterised by a higher accumulation of fat around the heart when compared to control groups [14, 15]. In the present study, we analyzed PERI, which is the sum of EPI and paracardial adipose tissue, as previously realized in earlier studies [10, 19, 22], while the separation of these two fat depots was especially in lean subjects not possible. Thus, we could not find significant differences in the amount of PERI between women with PCOS, defined by the NIH and the ROT criteria and the control subjects. An analysis of the whole PCOS population presented a 24% higher amount of PERI, when compared to the controls. Therefore, the lack of significance in the present study could have occurred due to the inequality and because of the lower number in the PCOS group and the control group and the differences in the mean BMI. An additional reason for the differences in the results compared to the studies of Aydogdu et al. [15] and Sahin et al. [14] could be explained by the implementation of different techniques in the measurement of EPI/PERI. The other studies measured the amount of EPI with echocardiography [14, 15], while we used the gold standard, the cardiac magnetic resonance imaging, for the analyzation of PERI [29]. Although we could not find significant differences in the amount of cardiac fat depots, PERI crystallized to be an interesting aspect in the carbohydrate metabolism especially in women with PCOS in the present study. We observed with a detailed assessment of glucose metabolism that only in PCOS women, who were characterised by a more pronounced insulin resistance when compared to the control group, PERI is positively related to parameters of glucose metabolism. These results are in line with prior reports showing that the increase of adipose tissue around the heart is related to disturbances of glucose metabolism [10, 12, 17, 18, 30], which concluded their results based upon mixed study populations. Controversial data about the relationship of EPI with parameters of glucose metabolism in the specific population of women with PCOS exist so far [11, 13–15]. This could possibly be the case because the assessment of glucose metabolism in the existing studies in women with PCOS was done with blood samples under fasting conditions and not with a detailed examination of glucose metabolism, such as the OGTT. A study group, investigating women with PCOS (mean BMI of 32 kg/m^2^) with blood samples under fasting conditions, demonstrated that EPI is related to fasting insulin and to HOMA-IR but not to glucose levels in women with PCOS [14]. These results are not at all in line with the results of the present study, demonstrating that there is a relationship of PERI with fasting glucose but not fasting insulin levels. However, the missing association of HOMA-IR with PERI in the present study could be caused by the differences in the mean BMI, when compared to the study group of Sahin et al. [14]. Nevertheless, the implementation of the OGTT in the present study allowed us to investigate the glucose metabolism in more detail, and we could additionally show that the increase of PERI in PCOS women is related to dynamic glucose and insulin levels and HbA1c levels and to inauspicious changes in beta cell function and insulin sensitivity. Thus, by splitting the PCOS cohort according to the NIH and ROT criteria, especially the relationship of PERI with fasting and dynamic glucose levels in the NIH group remained significant. However, in the ROT group, only the relationship of PERI with fasting plasma glucose levels could be observed. Therefore, these differences could be explained not only by the lower sample size in the ROT group but also by the differences in the waist circumference between the two study groups. In addition, it has to be mentioned that the different diagnostic criteria for PCOS of the NIH and the ROT may describe different phenotypes of PCOS. Thus, the fact that EPI is an endocrine active organ that expresses antidiabetogenic factors, such as adiponectin yet only under physiological conditions [16, 28], could play a major role. Studies showed that there is an inverse relationship of PERI with the levels of adiponectin [10, 31]. This could also be observed in the present study in the total group of women with PCOS. Thus, we also found a negative relationship of adiponectin with insulin resistance. So, the insufficient expression of antidiabetogenic factors by PERI under pathological conditions, such as in women with PCOS, seems to be related to the regulation of glucose metabolism in this specific population. The impression of a relationship of factors expressed by the adipose tissue around the heart and metabolic disturbances in PCOS could be justified by the fact that women with PCOS have lower levels of adiponectin compared to control subjects [15, 32]. Therefore, in addition to a control group, it is important to categorize patient groups according to various metabolic diseases, which enable clear assumptions according to the underlying pathophysiological mechanisms of PERI. The antidiabetogenic effect of PERI under physiological conditions is an interesting topic and not only the expression of adipokines but also the expression of other factors could be related to the regulation of glucose metabolism in PCOS. We observed a significant positive relation of PERI with prolactin levels in the control group of the present study. There is a link between the levels of prolactin and disturbances of glucose metabolism. Studies have shown that prolactin receptors are expressed by adipose tissue in humans [33] and that lower levels of prolactin are related to disturbances of glucose metabolism [34] whereas physiologically increased levels of prolactin have shown to have beneficial effects on glucose metabolism [35]. Further studies would therefore be necessary in order to investigate if there is a relation of the expression of prolactin by PERI with protective effects on glucose metabolism in healthy control subjects. Interestingly, the present study demonstrated that the amount of fat depots surrounding the heart is related to disturbances of glucose metabolism in women with PCOS; however, intramyocardial fat content is not. MYCL, the amount of fat in the heart, has been investigated in detail previously. Study groups demonstrated in different patient cohorts, including patients possibly having or not having diabetes [18] or patients with the metabolic syndrome [36] and also metabolically healthy subjects [19], that there is not a relationship of the amount of MYCL with parameters of glucose metabolism. In the present study, neither the control subjects nor the high-risk population of women with PCOS present a relation of MYCL with parameters and indices of the glucose metabolism. Furthermore, the entire study population was additionally analyzed, yet no relationship could be evaluated. Victor et al. hypothesized that insulin resistance in PCOS is related to the inflammatory status [37] and it has been demonstrated that PERI expresses proinflammatory markers [28]. In line with the reported results of Cakir et al. [13], we also observed a positive relation of PERI with the proinflammatory hs-CRP in women with PCOS, portraying that PERI is also related to the cardiovascular risk in women with PCOS. Besides the already discussed effects of PERI amongst pathological conditions, the fat content around the heart could be accompanied by dyslipidemia. Marchington et al. observed that the expression of free fatty acids was two times higher in EPI compared to other fat depots [38]. This allows to assume that there is an increased action of lipolytic activity [31]. Therefore, our observation that an increased amount of PERI is related to inauspicious concentrations of lipid parameters, such as LDL cholesterol or HDL cholesterol in women with PCOS, is in line with prior reports [31]. On the contrary, we observed a significant inverse relationship of PERI with triglyceride levels in the control group. Conclusively, physiologically increased levels of PERI in metabolically healthy subjects could have a positive influence on lipid parameters. Our results, which showed that PERI is related to the parameters of body composition (BMI and waist circumference) in the specific population of women with PCOS as well as in control subjects and in the whole study population, are in line with earlier studies [8, 19]. We also observed a positive relation of PERI with adipose tissue in the liver similar to prior studies [39].

## 5. Limitations

Our study has limitations and strengths. A limitation of the present study is the fact that the number of participants in the PCOS and the control group is unequal. A further limitation occurred, due to differences in the mean BMI, between women with PCOS and the healthy control group. In addition, 10 women of the control group were taking hormonal contraceptives, which could influence the results. Thus, we can also report about strengths of the present study. Therefore, the pericardial fat content was analyzed with the gold standard, the magnetic resonance spectroscopy. A further strength of the study is the assessment of glucose metabolism by using a dynamic test, the OGTT, which allowed us to analyze the glucose metabolism in more detail.

## 6. Conclusion

We can conclude that PERI, but not MYCL, is related to metabolic disturbances in women with PCOS and that it is important to categorize different patient groups in order to get conclusive results about the influence of PERI on carbohydrate metabolism. However, larger and longitudinal studies would be necessary to prove the results of the present study.

## Figures and Tables

**Table 1:** Basic characteristics of the study population.

	PCOS total (*n* = 48)	PCOS-NIH (*n* = 31)	PCOS-ROT (*n* = 17)	Controls (*n* = 20)	*p* value
Age (years)	25 (21–30)	25 (21–28)	24 (22–32)	23 (23–25)	0.475
BMI (kg/m^2^)	24 (22–30)^§^	25 (23–30)^§^	23 (21–30)	21 (20–24)	**0.005**
Waist (cm)	84 (74–93)	88 (80–96)^§∗^	75 (69–84)	78 (75–85)	**0.003**
RRsys (mmHg)	114 (105–120)	113 (107–121)	115 (104–122)	116 (111–126)	0.487
RRdia (mmHg)	72 (65–79)	72 (66–79)	70 (63–78)	73 (63–76)	0.739
HF (beats per minute)	73 (64–80)	74 (66–85)	72 (64–80)	74 (63–77)	0.698
Total cholesterol (mg/dl)	177 (153–201)	178 (153–203)	175 (162–198)	170 (158–180)	0.435
HDL-cholesterol (mg/dl)	58 (46–67)^§^	57 (44–65)^§^	60 (47–73)	68 (60–78)	**0.019**
LDL-cholesterol (mg/dl)	100 (74–125)	99 (73–128)	102 (76–121)	81 (70–87)	0.074
Triglycerides (mg/dl)	75 (60–87)^§^	72 (59–84)^§^	84 (63–88)	99 (78–110)	**0.030**
Matsuda index	5.5 (3.4–8.8)^§^	5.1 (3.6-7.1)^§^	6.2 (3.0–11.0)^§^	8.9 (5.5–13.3)	**0.004**
HOMA-IR	1.6 (1.0–2.4)^§^	1.6 (1.1-2.4)^§^	1.7 (0.8–2.4)	0.9 (0.5–1.7)	**0.024**
Insulinogenic index	1.1 (0.6–2.0)	1.1 (0.6–2.2)	1.1 (0.7–1.6)	0.9 (0.6–1.3)	0.582
Disposition index (IGI^∗^ISI)	5.4 (3.2–9.0)	5.3 (3.2–9.8)	5.7 (2.9–8.8)	6.4 (4.7–11.5)	0.278
OGIS	494 (446–542)	481 (449–542)	503 (417–544)	537 (499–549)	0.179
MYCL (%)	0.48 (0.27–1.07)	0.49 (0.28–1.36)	0.47 (0.22–0.87)	0.50 (0.23–0.75)	0.801
PERI (cm^2^)	11.22 (7.91–16.61)	11.65 (7.60–17.34)	10.67 (8.05–16.20)	8.52 (7.07–12.04)	0.231

PCOS total: sum of PCOS-NIH and PCOS-ROT; PCOS-NIH: women with PCOS defined according to the criteria of the National Institute of Health; PCOS-ROT: women with PCOS defined according to the Rotterdam criteria; BMI: body mass index: waist: waist circumference; RRsys: systolic blood pressure; RRdia: diastolic blood pressure; HF: heart frequency; HDL-cholesterol: high-density lipoprotein cholesterol; LDL-cholesterol: low-density lipoprotein cholesterol; MYCL: intramyocardial fat; PERI: pericardial fat. ^§^Versus controls: *p* < 0.05; ^∗^PCOS-NIH versus PCOS-ROT: *p* < 0.05.

**Table 2:** Correlation analyses of pericardial fat (PERI) with parameters and indices of glucose metabolism in women with PCOS and healthy control subjects.

PERI	PCOS total		PCOS-NIH		PCOS-ROT		Controls	
	Rho	*p*	Rho	*p*	Rho	*p*	Rho	*p*
Disposition index (IGI∗ISI)	**−0.346**	**0.027**	−0.295	0.162	−0.453	0.068	0.403	0.087
OGIS	**−0.389**	**0.012**	−0.359	0.085	−0.471	0.057	−0.192	0.432
Matsuda index	−0.242	0.132	−0.124	0.574	−0.360	0.155	0.264	0.274
HOMA-IR	0.273	0.076	0.197	0.334	0.311	0.224	−0.268	0.254
Insulinogenic index	−0.129	0.409	−0.138	0.502	−0.108	0.680	0.010	0.967
HbA1c	**0.486**	**0.001**	**0.573**	**0.003**	0.442	0.076	−0.384	0.095
Fasting plasma glucose	**0.682**	**<0.001**	**0.737**	**<0.001**	**0.630**	**0.007**	0.128	0.591
Glucose 30′	**0.449**	**0.003**	**0.441**	**0.024**	0.469	0.058	−0.238	0.313
Glucose 60′	**0.505**	**0.001**	**0.596**	**0.001**	0.363	0.152	0.052	0.828
Glucose 90′	**0.389**	**0.012**	**0.448**	**0.028**	0.305	0.234	−0.187	0.444
Glucose 120′	**0.377**	**0.013**	**0.389**	**0.050**	0.241	0.352	−0.094	0.693
Insulin 0′	0.193	0.214	0.112	0.587	0.262	0.309	−0.269	0.252
Insulin 60′	**0.306**	**0.046**	0.260	0.199	0.365	0.149	0.182	0.442
C-peptide 0′	0.206	0.185	0.021	0.919	0.395	0.117	−0.223	0.345

**Table 3:** Correlation analyses of pericardial fat (PERI) with lipid parameters, ectopic lipids, hormones, and anthropometric data in women with PCOS and healthy control subjects.

PERI	PCOS total		PCOS-NIH		PCOS-ROT		Controls	
	Rho	*p*	Rho	*p*	Rho	*p*	Rho	*p*
TG	0.156	0.318	0.195	0.339	0.054	0.837	**−0.512**	**0.021**
HDL	**−0.343**	**0.024**	**−0.460**	**0.018**	0.015	0.955	0.266	0.257
LDL	**0.381**	**0.012**	**0.530**	**0.005**	0.086	0.743	0.135	0.569
BMI	**0.343**	**0.024**	**0.475**	**0.014**	0.164	0.529	**0.506**	**0.023**
Waist	**0.377**	**0.014**	**0.578**	**0.002**	0.084	0.750	**0.509**	**0.022**
Liver fat	**0.464**	**0.002**	**0.598**	**0.002**	0.309	0.228	0.422	0.072
CRP	**0.328**	**0.032**	**0.517**	**0.007**	−0.006	0.981	−0.440	0.052
hsCRP	**0.337**	**0.031**	**0.489**	**0.015**	0.108	0.679	−0.407	0.075
Cortisol	−0.173	0.293	−0.326	0.112	0.205	0.483	−0.410	0.081
Prolactin	−0.117	0.462	−0.026	0.900	−0.356	0.176	**0.577**	**0.010**
Bioestradiol	−0.133	0.408	−0.337	0.100	0.108	0.692	0.228	0.395
Biotestosterone	0.073	0.645	−0.063	0.761	0.380	0.147	0.100	0.675
Progesterone	−0.091	0.570	−0.115	0.577	−0.064	0.820	0.081	0.734

TG: triglycerides; HDL: high-density lipoprotein cholesterol; LDL: low-density lipoprotein cholesterol; BMI: body mass index; waist: waist circumference; hsCRP: high sensitive C-reactive protein; Bioestradiol: bioavailable estradiol; Biotestosterone: bioavailable testosterone.

## Supplementary Materials

Supplementary Materials Table 1: linear regression models in the PCOS total group.
