# Supplementary material for: Pericardial Fat Relates to Disturbances of Glucose Metabolism in Women with the Polycystic Ovary Syndrome, but Not in Healthy Control Subjects
Source: Int J Endocrinol. 2018 Aug 7;2018:5406128. doi: 10.1155/2018/5406128 (PMC6109482; doi:10.1155/2018/5406128)
Supplement: Supplementary Materials — Table 1: linear regression models in the PCOS total group. [file 5406128.f1.docx]

**Supplementary table 1:** Linear regression models in the PCOS-totals group

|  |  | |  |  |  |
| --- | --- | --- | --- | --- | --- |
| **Independent variables** | **R** | **E** | **Beta** | **T** | **p-value** |
| **Dependent variable: HbA1c** |  |  |  |  |  |
| Age | 0.000 | 0.008 | -0.006 | -0.040 | 0.968 |
| BMI | 0.012 | 0.009 | 0.202 | 1.305 | 0.200 |
| Pericardial fat | 0.014 | 0.006 | 0.370 | 2.211 | **0.033** |
| **Dependent variable: fasting plasma glucose** |  |  |  |  |  |
| Age | -0.298 | 0.168 | -0.235 | -1.774 | 0.084 |
| BMI | 0.252 | 0.202 | 0.161 | 1.243 | 0.221 |
| Pericardial fat | 0.651 | 0.142 | 0.647 | 4.576 | **<0.001** |
| **Dependent variable: glucose 30´** |  |  |  |  |  |
| Age | -0.447 | 0.642 | -0.110 | -0.697 | 0.490 |
| BMI | 0.717 | 0.774 | 0.143 | 0.926 | 0.360 |
| Pericardial fat | 1.303 | 0.544 | 0.405 | 2.394 | **0.022** |
| **Dependent variable: glucose 60´** |  |  |  |  |  |
| Age | -0.657 | 0.952 | -0.104 | -0.689 | 0.495 |
| BMI | 1.363 | 1.149 | 0.175 | 1.186 | 0.243 |
| Pericardial fat | 2.272 | 0.807 | 0.456 | 2.813 | **<0.001** |
| **Dependent variable: glucose 90´** |  |  |  |  |  |
| Age | 0.974 | 0.808 | 0.193 | 1.206 | 0.235 |
| BMI | 1.689 | 0.955 | 0.267 | 1.769 | 0.085 |
| Pericardial fat | 0.911 | 0.684 | 0.224 | 1.332 | 0.191 |
| **Dependent variable: glucose 120´** |  |  |  |  |  |
| Age | 0.674 | 0.627 | 0.169 | 1.074 | 0.289 |
| BMI | 1.740 | 0.757 | 0.352 | 2.299 | **0.027** |
| Pericardial fat | 0.293 | 0.532 | 0.093 | 0.552 | 0.584 |
| **Dependent variable: Disposition index (IGI*ISI)** |  |  |  |  |  |
| Age | -0.010 | 0.225 | -0.008 | -0.045 | 0.965 |
| BMI | -0.095 | 0.265 | -0.060 | -0.358 | 0.722 |
| Pericardial fat | -0.284 | 0.190 | -0.277 | -1.494 | 0.144 |
| **Dependent variable: OGIS** |  |  |  |  |  |
| Age | 3.975 | 1.991 | 0.291 | 1.996 | 0.053 |
| BMI | -6.302 | 2.354 | -0.369 | -2.677 | **0.011** |
| Pericardial fat | -4.900 | 1.686 | -0.446 | -2.906 | **0.006** |

____________________________________________________________________________________________________
